# Standardization of ^63^Ni by 4πβ Liquid Scintillation Spectrometry With ^3^H-Standard Efficiency Tracing

**DOI:** 10.6028/jres.102.031

**Published:** 1997

**Authors:** B. E. Zimmerman, R. Collé

**Affiliations:** National Institute of Standards and Technology, Gaithersburg, MD 20899-0001

**Keywords:** beta-counting, CIEMAT/NIST Method, liquid scintillation, nickel-63, radioactivity, standards

## Abstract

The low energy (*E_β_*_max_ = 66.945 keV ± 0.004 keV) *β*-emitter ^63^Ni has become increasingly important in the field of radionuclidic metrology. In addition to having a low *β*-endpoint energy, the relatively long half-life (101.1 a ± 1.4 a) makes it an appealing standard for such applications. This paper describes the recent preparation and calibration of a new solution Standard Reference Material of ^63^Ni, SRM 4226C, released by the National Institute of Standards and Technology. The massic activity *C*_A_ for these standards was determined using 4*πβ* liquid scintillation (LS) spectrometry with ^3^H-standard efficiency tracing using the CIEMAT/NIST method, and is certified as 50.53 kBq ·g^−1^ ± 0.46 Bq · g^−1^ at the reference time of 1200 EST August 15, 1995. The uncertainty given is the expanded (coverage factor *k* = 2 and thus a 2 standard deviation estimate) uncertainty based on the evaluation of 28 different uncertainty components. These components were evaluated on the basis of an exhaustive number (976) of LS counting measurements investigating over 15 variables. Through the study of these variables it was found that LS cocktail water mass fraction and ion concentration play important roles in cocktail stability and consistency of counting results. The results of all of these experiments are discussed.

## 1. Introduction

In recent years, ^63^Ni has become an increasingly important radionuclide in the fields of analytical chemistry and radionuclidic metrology. Because it is the product of neutron capture by ^62^Ni, which is found in steel used in the construction of nuclear reactor facilities, it is of considerable interest to the nuclear power community. Because it has a relatively long half-life (101.1 a ± 1.4 a) [1] and is a pure *β*-emitter with a low end-point energy (66.945 keV ± 0.004 keV) [2], it is becoming an increasingly used radionuclide in studies involving 4*πβ* liquid scintillation spectrometry (LS). These physical properties make subtle effects associated with this technique more apparent than would be with other, higher-energy, *β*-emitters. This paper describes the preparation and calibration of a solution Standard Reference Material (SRM) of ^63^Ni recently issued by the National Institute of Standards and Technology.

## 2. Experimental

### 2.1 Policy on Treatment of Measurement Uncertainties

All evaluation of measurement uncertainties throughout this work follow accepted conventions used by the NIST Radioactivity Group and are in concordance with those recommended by the principal metrology standards organizations [3]. All individual uncertainty components are given as estimated experimental standard deviations (or standard deviations of the mean, if appropriate), or quantities assumed to correspond to standard deviations regardless of the method used to evaluate their magnitude. Unless explicitly stated, all uncertainties cited in this paper are “standard uncertainties,” corresponding to one uncertainty interval. One particular exception is the uncertainty reported for the certified massic activity^1^ of the ^63^Ni standard, which is given as an “expanded combined standard uncertainty.” In accordance with NIST policy [4], the combined standard uncertainty (calculated by combining the individual uncertainty components in quadrature) is multiplied by a “coverage factor” of *k* = 2 to obtain an “expanded uncertainty” assumed to give an uncertainty interval having a confidence level of 90 % to 95 %.

### 2.2 Preparation of Master Solution and SRM Ampoules

**Fig. 1:**
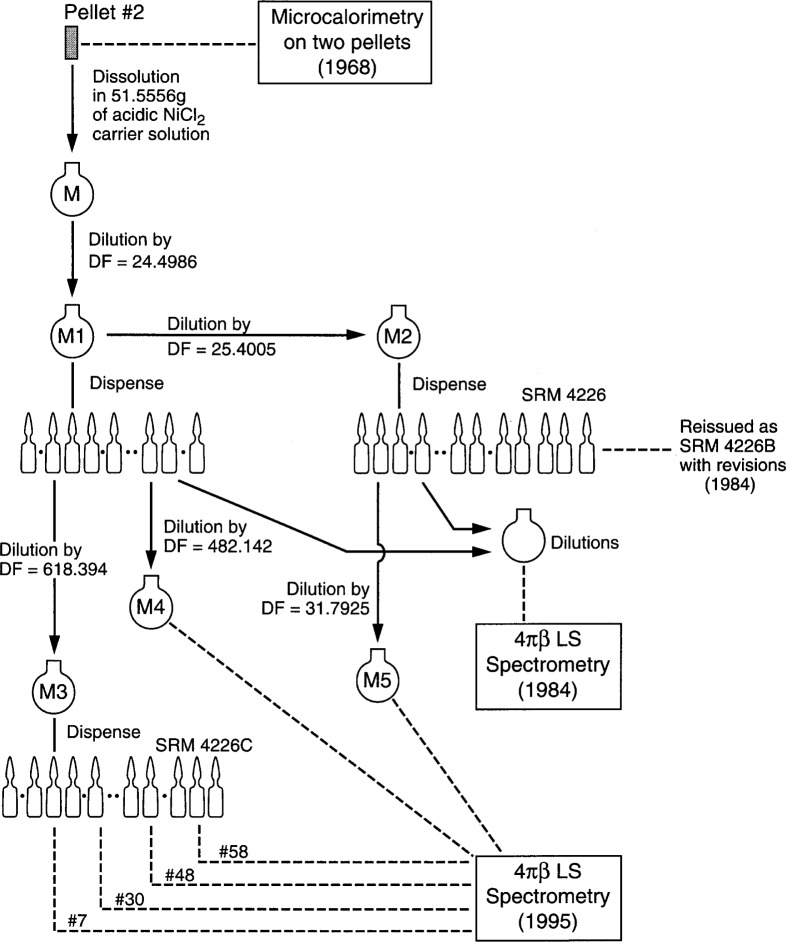
Schematic diagram of preparation of and relationships between the NBS/NIST SRM4226 series of ^63^Ni solution standards.

The origin of the solution used to prepare the present SRM is depicted in Fig. 1. The original solution (labeled “M1” in the figure) was prepared in 1968 by dissolving a pellet of ^63^Ni (produced by the neutron-activation of ^62^Ni, supplied by Oak Ridge National Laboratory) in a solution containing concentrated HCl and concentrated HNO_3_. The resulting solution (labeled “M”) was then diluted with acidic NiCl_2_ solution by a factor of 24.4986 (labeled “M1”). A portion of this subsequent solution was diluted further with acidic NiCl_2_ solution by a factor of 25.4005 (labeled “M2” in the figure). This solution was calibrated and issued as SRM 4226, and subsequently re-calibrated in 1984 and issued as SRM4226B.

In order to prepare the present solutions, an aliquant of solution from an ampoule containing solution from M1 was gravimetrically transferred (using a polyethylene aspiration pycnometer) to a mixing bottle containing a carrier solution of 98 mg of Ni^+2^ per gram of solution in nominally 1 mol · L^−1^ HCl (NIST high-purity grade) to give a dilution factor of 618.394 relative to the M1 solution. An automatic dispenser was used to fill 60 borosilicate glass ampoules. The average solution mass per ampoule was determined from the mass difference (full and empty) for 14 ampoules chosen at random from the set of 60 ampoules and was determined to be 5.080 g ± 0.002 g, where the uncertainty is the standard deviation for dispensing repeatability into those 14 ampoules.

Based on these mass measurements and the known dispensing volume, the density of the solution was found to be 1.016 g · mL^−1^ ± 0.002 g · mL^−1^. Three additional density measurements were made using 50 mL and 25 mL volumetric flasks. These values, combined with the average obtained from the dispensings, resulted in an average of 1.014 g · mL^−1^ ± 0.004 g · mL^−1^, where the cited uncertainty is the standard uncertainty and is equal to the standard deviation of the four density values combined in the average.

After filling, the ampoules were flame-sealed and autoclaved. Four ampoules (numbered 7, 30, 48, and 58 by order of filling) were then chosen at random from the set of 60 ampoules to perform the calibration experiments.

### 2.3 Preparation of Liquid Scintillation Sources

#### 2.3.1 Methods and Materials

All additions of aliquants in the experiments described below were performed gravimetrically using polyethylene aspiration-type pycnometers. Mass measurements were performed with several (depending upon the mass ranges) single-pan suspension microbalances with internal reference weights. For mass measurements involving items with masses greater than 20 g, a 200 g single-pan suspension balance with internal weights was employed. Masses above 100 g were determined with a 2 kg single-pan suspension balance with internal balance weights. The relative uncertainty of any mass determination after application of the appropriate air-buoyancy corrections is estimated to be ± 0.05 %. Gravimetric additions of radioactive solutions (^63^Ni and ^3^H) were performed using a microbalance.

The liquid scintillation (LS) cocktails were counted using two commercially-available LS spectrometers.^2^ The first was a Packard TriCarb2500TR LS analyzer, which employs two photomultiplier tubes (PMTs) in coincidence mode, linear signal amplification for pulse height analysis of spectra, and an external ^133^Ba *γ*-ray source for quench determinations. The quench-indicating parameter (QIP) was *tSIE*, which uses a proprietary algorithm to obtain a mathematical transform of the Compton spectrum produced by the *γ*-rays of the external source, from which the *tSIE* is calculated by a mathematical combination of the coefficients of the transform. The second system was a Beckman LS7800 LS counter equipped with two Hamamatsu R331-05 PMTs, also operated in coincidence mode, but with logarithmic signal amplification. The QIP was the Horrocks *H*#, which was determined with an externally-placed ^137^Cs *γ*-ray source and which measures the shift (in channels) of the edge of the Compton distribution of electrons produced by the external source of a quenched cocktail relative to an “unquenched” cocktail.

The ^3^H standard solutions were gravimetric dilutions of NIST SRM 4927E, which was primarily calibrated in September 1961 by internal gas counting [5] and recertified in 1991 [6].

All of the LS cocktails were prepared using glass vials of nominal 22 mL volume fitted with cork-backed, aluminum foil-lined plastic caps. The cocktails were generally prepared in the following steps:

1. One of three commercially prepared scintillation fluids (Beckman Ready-Safe, Packard Ultima-Gold AB, or Packard Insta-Gel XF) were dispensed into the vials using a graduated, manual dispensing pipette. An average mass of added scintillator was obtained from mass differences between empty and full vials of several vials chosen at random from each set prepared. For some of the experiments outlined below, the mass of scintillator added to each individual vial was determined after volumetric addition.
2. Any additional water or Ni^+2^ carrier solution was then added, either by determining the average mass of added drops or by gravimetric determination of each separate addition.
3. Some experiments utilized an induced-quenching agent to provide the quenching range necessary to develop a quench-correction curve. For those studies, either nitromethane (CH_3_NO_2_), or a 10 % (by volume) solution of nitromethane in ethanol, was added dropwise to the cocktail.
4. Radioactive solution was gravimetrically added to the cocktail in such a way as to avoid contact with the walls of the vial prior to mixing to prevent losses due to absorption of material into the glass.
5. The vials were capped and agitated for several minutes to ensure mixing of all of the added components of the cocktail. The vials were placed in one of the two spectrometers and allowed to dark-adjust for at least 30 min. If the vials were to be counted on a second spectrometer, they were re-agitated and dark-adjusted after transfer to the spectrometer and prior to counting.

The typical counting arrangement consisted of between 4 and 10 closely matched (in terms of composition, and thus quenching) pairs of ^3^H and ^63^Ni cocktails with varying quench and two to four background blanks containing distilled water and (in most experiments) Ni^+2^ carrier solution. The ^3^H and ^63^Ni cocktails were alternately counted in sequence of increasing quench. The backgrounds were positioned at equal intervals throughout the counting series. Count times were usually 20 min livetime and each series was generally counted for 6 cycles. No internal instrumental corrections of any kind, other than livetime, were used. Deadtime losses for any counting source used in this work were less than 3 %.

Impurity levels of *γ*-emitting radionuclides were determined using an ampoule containing solution from M1 (prior to dilution) with a calibrated HPGe *γ*-ray spectrometry system.

#### 2.3.2 Preliminary Experiments

The overall goal of the preliminary experiments was to identify and quantify as many of the sources of uncertainty as possible while making a large number of measurements of the massic activity. To this end, 33 matched ^3^H and ^63^Ni cocktail pairs (plus background blanks) were prepared over the course of 22 experiments. An additional set of experiments involving total cocktail mass (volume) effects was performed and has been previously described [7]. The various LS cocktail compositions used in the calibration experiments are summarized in Table 1. Details of the experiments for each set of cocktails are presented below.

##### Experiments “A” to “H”

The cocktails prepared for these eight trials were used to study the differences between two of the scintillators, the two spectrometers, and the effects of cocktail age in applying the subsequent efficiency-tracing method. The first set of counting sources used in trials “A” through “D” and “H” consisted of five pairs of matched ^3^H and ^63^Ni cocktails having 15 g of Ready Safe scintillatant. Five additional pairs were prepared with Ultima Gold AB. The average mass of the added scintillatant was obtained from mass differences between empty and filled LS vials for each of three Ready Safe and Ultima Gold cocktails. Three additional cocktails of either Ready Safe or Ultima Gold were also were also prepared for each set to serve as background blanks. These blanks had compositions that were similar to the ^63^Ni and ^3^H cocktails.

The amount of gravimetrically-added radioactive ^3^H and ^63^Ni solutions was nominally 15 mg. The ^63^Ni solution was from ampoule 30. About 15 mg of Ni^+2^ carrier was added to each of the six background blanks. Between 0 mg and 400 mg of the diluted nitromethane was added dropwise as an induced quenching agent. Amounts were added to the backgrounds such that the amount added covered the 0 mg to 400 mg range used in the ^3^H and ^63^Ni cocktails.

The two series of cocktails were counted alternately between the two spectrometers for 6 cycles of 20 min livetime. The Ultima Gold cocktails (series E, F, G) were counted over the course of 9 d, while those with Ready Safe were counted over the course of 86 d.

##### Experiments “I” and “J”

These experiments were intended to investigate any influence on the efficiency tracing due to changes in the amount of ^63^Ni solution as it might affect both the activity and Ni^+2^ loading. Possible variations due to use of a different ^63^Ni ampoule were also studied. The amount of water added to the cocktails was also increased above the first set of cocktails from an aqueous mass fraction *f* of 0.001 to *f* 0.03 in order to determine what effect, if any, *f* would have on the efficiency-traced massic activity.

A total of 21 cocktails were prepared by initially adding approximately 14.5 g of Ultima Gold and 0.5 g of distilled water to each of the vials. The average masses of dispensed scintillator and water were obtained from mass differences of five randomly chosen vials. Between 40 mg and 50 mg of ^63^Ni solution from ampoule 7 were then gravimetrically added to nine of the vials. Nine other vials had 30 mg to 40 mg of the diluted ^3^H water standard gravimetrically added to them. Three background blanks were prepared by gravimetric additions of 20 mg to 30 mg of the Ni^+2^ ion carrier to the scintillant. The quench was varied in the ^3^H and ^63^Ni cocktails by the dropwise addition of zero to 400 mg of the diluted nitromethane. No quencher was added to the backgrounds. All of the cocktails were counted for 20 min livetime over at least 5 cycles in both the Packard and Beckman spectrometers.

##### Experiments “K” and “L”

The third series of cocktails used in these two preliminary experiments was designed in such a way as to allow investigations of the dependency on spectrometer used, the effect of using a lower total cocktail volume, differences between ampoules, variations in aqueous mass fraction in the cocktail, and the effect of using a very large quenching range on the efficiency-traced activity. They were also the first experiments of this study in which the ^3^H and ^63^Ni cocktails were prepared so as to be very closely matched in terms of composition. A total of 14 sources were prepared—six each of ^3^H and ^63^Ni, and two backgrounds. The sources were prepared with 10 g of Ready Safe added volumetrically to each vial, with an average mass obtained by mass differences for three randomly chosen vials. Between 15 mg and 60 mg of the ^63^Ni solution from ampoule 58 were gravimetrically added to the ^63^Ni cocktails. Between 15 mg and 80 mg of ^3^H were gravimetrically added to the ^3^H cocktails. In order to ensure that the cocktails were closely matched, nickel carrier solution was added to the ^3^H cocktails in amounts such that the Ni^+2^ ion number concentration in both the ^3^H and ^63^Ni cocktails were matched. In a similar fashion, distilled water was added to the ^63^Ni cocktails to account for the additional water present in the ^3^H cocktails from tritiated water. A large quenching range was obtained for the cocktails by the dropwise addition of undiluted nitromethane. Two background blanks were prepared so as to cover the quenching range of the ^3^H and ^63^Ni cocktails, with the first cocktail containing nominally 15 mg of Ni^+2^ carrier (gravimetrically added) and no added quencher, and the second containing nominally 60 mg of Ni^+2^ ion carrier and four drops of undiluted nitromethane.

The vials were counted in the Beckman for 7 cycles of 20 min livetime counts (trial “K”), then transferred to the Packard for 6 cycles of 20 min livetime counts (trial “L”).

##### Experiments “M” and N”

These experiments provided a confirmation of the present calibration by comparisons with those for NIST SRM 4226 and SRM 4226B. Largely, they could be used to confirm the dilution factors of the previous two ^63^Ni SRMs. Alternatively, they could be considered to be a check of the chemical stability of the solutions over 27 years. Because solution M1 (see Fig. 1) was of such high activity, it was necessary to dilute the solution from one of the ampoules of this batch to allow it to be counted in the LS spectrometers. One such ampoule was obtained and gravimetrically diluted with Ni^+2^ carrier solution by a factor of *DF* = 482.142 and labeled “M4.” Likewise, an ampoule containing solution M2 (the source of the solutions for SRMs 4226 and 4226B) were obtained. This was also diluted, but by a factor of only 31.792, and was labeled “M5.”

Five LS sources were prepared from each of the two ^63^Ni solutions (“M4” and “M5”), as well as five ^3^H sources and two background blanks. A volume of 10 mL of Ready Safe was volumetrically added to the vials, and the average mass of added scintillant was obtained by mass differences between two randomly chosen vials. A series of cocktails with increasing activity for the two ^63^Ni solutions as well as the ^3^H was obtained by the gravimetric addition of nominally 15 mg to 65 mg of the active solution to their respective set of LS vials. The compositions of the cocktails in each series were matched to each other by the addition of ^63^Ni carrier and distilled water to the respective ^3^H and ^63^Ni cocktails as described above. The quench was varied by the dropwise addition of zero to 0.06 g of the undiluted nitromethane. The backgrounds were prepared so as to reflect the two extremes of cocktail composition in the sets of ^63^Ni cocktails; that is, one vial contained nominally 15 mg each of the distilled water and Ni^+2^ carrier and no added quencher, while the other had nominally 70 mg each of the distilled water and Ni^+2^ carrier and 4 drops of the undiluted nitromethane.

The cocktails were counted in the Beckman spectrometer for 7 cycles of 20 min counts.

##### Experiments “O” To “V”

Previous and parallel studies of volume effects in ^3^H-standard efficiency tracing of ^63^Ni and ^36^Cl indicate [7, 8, 9] that changes or mismatches in total cocktail mass (or, alternatively, in volume) introduce an additional uncertainty into the determination of massic activity. With this in mind, the experiments for this series of cocktails were designed so as to keep the total cocktail mass as constant as possible while altering the water fraction. To do this, two sets of vials, one containing 8.8 g to 10 g (gravimetrically dispensed) of Ready Safe, and the second set containing the same amounts of Ultima Gold, were prepared for both the ^3^H and ^63^Ni cocktails series. The water fractions covered the range from *f* ≈ 0.003 to *f* ≈ 0.125. Two background blanks containing distilled water and nominally 15 mg of Ni^+2^ carrier were prepared in similar fashion to include the two extremes of cocktail composition (aqueous fraction). All changes in quenching were due solely to the amount of water in the cocktails—no additional quenching agent was added. Approximately 15 mg of either the ^3^H or the ^63^Ni (from ampoule 30) were added to the vials.

The cocktails were counted using both spectrometers either in 3 cycles of 30 min counts or 5 cycles of 20 min each. Some degree of information regarding cocktail stability and other efficiencies as a function of time was obtained by repeated counting of all cocktails in both spectrometers over the course of 8 d.

#### 2.3.3 “Final” Calibration Experiments

Based on the data gathered from the above experiments, the final calibration experiments were designed to isolate the effects of water fraction and the addition of Ni^+2^ carrier on the measured massic activity. It was also possible with these experiments to look at the relative quenching effects of the Ni^+2^ carrier and the diluted and concentrated nitromethane solutions. A final set of cocktails was prepared to look into effects of water fractions in cocktails forming gels by looking at the massic activities determined in both the “normal” and “gel” phases.

##### Experiments “W” to “Z”

These experiments were designed to look more closely at water fraction and cocktail matching effects and to study how these effects are altered by which scintillator is used. Solution M3 from still another ampoule was used to provide an additional check as to the consistency of the activity among the ampoules. The cocktails were prepared in a similar fashion to those in Sec. 2.2, except that the total cocktail mass was kept at 12 g and the aqueous fractions were higher (*f* = 0.08 to *f* = 0.20). Two sets of cocktails were prepared, one each for Ready Safe and Ultima Gold. The cocktail series for both sets included five ^63^Ni cocktails, five ^3^H cocktails, and two background blanks. Between 9.9 g and 11.3 g of scintillant were first gravimetrically added to each vial in the cocktail series. Nominally 15 mg of Ni^+2^ carrier were added dropwise to the ^3^H cocktails and to the background blanks. Additional water was gravimetrically added to all of the vials to bring the total cocktail mass in each to 12 g. Nominally 15 mg of either the ^3^H or ^63^Ni (from ampoule 48) were then added to the vials.

The backgrounds (four in all—two each of the Ready Safe and Ultima Gold) were made up so as to have the minimum and maximum water fractions of the ranges of the ^63^Ni and ^3^H cocktails. All quenching of the cocktails was due to the added water—no added imposed quencher was used.

The cocktails were counted for six cycles of 20 min each in both the Packard and Beckman.

##### Experiments “AA” to “AD”

The goal of this experiment was to investigate effects of added Ni^+2^ carrier to the solutions. To do this, two sets of cocktails were prepared for each nuclide. The first (for trials “AA” and “AC”) had a constant water fraction (nominally *f* = 0.1) and a constant total cocktail mass of 12 g. Approximately 10.5 g of Ultima Gold was gravimetrically dispensed into the scintillation vials (five each of ^63^Ni and ^3^H cocktails), followed by the gravimetric addition of 1.2 g of water. To these were gravimetrically added 15 mg of either the ^63^Ni or the ^3^H. Quench variation was achieved by the dropwise addition of undiluted nitromethane.

The second set of cocktails also maintained constant water fraction, but varied the contribution to the total water mass from the Ni^+2^ carrier solution. Four cocktails were prepared for both the ^3^H and ^63^Ni cocktails in a manner similar to that described before, with the variation being that the Ni^+2^ carrier added to both sets of cocktails accounted for 0 %, 25 %, 50 %, and 100 % of the total aqueous portion of the cocktail. No additional quenching agent was used—all quenching variations were accomplished by the varying amounts of Ni^+2^ carrier ions.

Two background blanks were prepared for this experiment with the same water fraction (*f* = 0.1), but with the Ni^+2^ carrier fraction equal to 25 % and 100 % of the total water fraction in the cocktail.

The cocktails were counted in both the Beckman and Packard spectrometers for 4 to 5 cycles of 20 min live-time counts.

##### Experiments “AE” to “AH”

Cocktails for these experiments utilized the scintillator Insta-Gel XF to look at water fraction effects in both the “normal” and “gel” phases of the scintillator. Between nominally 6.5 g to 12 g of Insta-Gel XF was gravimetrically dispensed into two sets of 8 vials, plus two background blanks. Masses of 15 mg of ^3^H solution and nominally 15 mg of the Ni^+2^ carrier were gravimetrically added to eight of the vials. The water fraction (and thus also the phase of the cocktail) was altered by the gravimetric addition of water such that all cocktails contained a total cocktail mass of 12 g. The ^63^Ni cocktails were prepared similarly, with solution from ampoule 7. No additional carrier, other than the amount needed to match the ^3^H cocktails to the ^63^Ni cocktails (≈ 15 mg), was added. The total aqueous fractions of the cocktails spanned the ranges *f* = 2 · 10^−5^ to *f* = 0.1 (“normal phase”) and *f* = 0.29 to *f* = 0.46 (“gel phase”).

The two blanks were prepared so that one had a composition in the “normal” phase (*f* = 0.08), and the other was in the gel phase (*f* = 33). Only distilled water was added to achieve the desired aqueous fractions in the cocktails.

The cocktails were counted in both the Beckman and Packard spectrometers for 4 cycles of 20 min livetime counts.

## 3. Results and Discussion

### 3.1 The CIEMAT/NIST^3^ ^3^H-Standard Efficiency Tracing Method

The CIEMAT/NIST 3H efficiency tracing method [10, 11, 12] is a protocol by which the LS counting efficiency for a cocktail of interest under known, varying quenching conditions is obtained by following the efficiency of a closely matched (in terms of cocktail composition) standard. Tritium is a good candidate for this standard because of its low *β*-energy, which makes it very sensitive to quenching effects.

In order to describe the overall efficiency of the counting system, a “figure of merit” *M* is employed. This parameter describes the energy (in keV) required to produce one photoelectron at the first dynode of the PMT. For a decay event with energy *E* from the *β*-spectral distribution, the fraction of energy lost due to heat absorption outside the solution (i.e., the container walls) is given by 1–*W*(*E*), while the fractional energy loss due to secondary interactions within the solution (“quenching”) is given by 1*–Q*(*E*). The energy remaining, and thus available to produce photoelectrons, is then *EQ*(*E*)*W*(*E*) and represents the unquenched energy of the decay event. The average number of photoelectrons produced at the first dynode of the PMT is then

$$\bar{n}=M^{-1}EQ\left(E\right)\;W\left(E\right).$$ (1)

For low energies (< 100 keV), *β*-particles with the distribution of these photoelectrons are assumed to follow Poisson statistics (at higher *β*-energies the distribution is normal). Thus the probability of detecting *x* photoelectrons from a mean number of photoelectrons $\bar{n}$ can be calculated by

$$P\left(x,\;n\right)=\frac{n^{-x}}{x!}\exp\left(-\bar{n}\right),$$ (2)

and the probability of detecting zero electrons (the “nondetection probability”) is just

$$P\left(0,\;\bar{n}\right)=\exp\left(-\bar{n}\right),$$ (3)

where the probability distributions are normalized to 1. The detection probability for a single PMT is then $\left(1-\exp\left(-\bar{n}\right)\right)$. Hence, for a two-PMT system in coincidence, the expected efficiency above the detection threshold is given by

$$\begin{array}{l} \hat{\varepsilon }\left(E\right)={\left\{1-\exp\left[-\bar{n}\right]\right\}}^{2}\\ \;={\left\{1-\exp\left[-M^{-1}EQ\left(E\right)\;W\left(E\right)\right]\right\}}^{2}. \end{array}$$ (4)

This efficiency, however, still only applies to events *above* the detection threshold. In order to include events below the threshold, an extrapolation to zero detection threshold must be made.

This is achieved in the CIEMAT/NIST method by relating this probabilistic efficiency $\hat{\varepsilon }$ to one that can be determined experimentally. The calculation model for this transformation is embodied in the code EFFY4, which is an updated and revised version of EFFY2 [13, 14]. The program calculates the efficiency for a given *M* by first calculating the shape of the *β*-spectrum for a particular nuclide through the Fermi distribution function *P*(*Z, E*) d*E* (not to be confused with the Poisson probability distribution *P*(*x*, *n*), given above). By comparing the number of particles emitted to the number of those that would most likely be detected, the efficiency can be calculated by the relationship

$$\hat{\varepsilon }\frac{\int_{0}^{E_{\max}}{\left\{1-\exp\left[-M^{-1}EQ\left(E\right)\right]\right\}}^{2}P\left(Z,\;E\right)dE}{\int_{0}^{E_{\max}}P\left(Z,E\right)dE}$$ (5)

where the integrals represent the total number of counts (numerator) or *β*-particles (denominator) over the energy from 0 keV to the *β*-endpoint.

In practice, the procedure consists of running EFFY4 to obtain a table of *M* vs *ε* [(accomplished by numerical integration of Eq. (5)] for each of the nuclides involved in the study, including the standard. The efficiency of the standard is then experimentally determined and a corresponding value of *M* is read from the table. The precision in the determinations is based on the step size between successive *M* -values used when running EFFY4. A fit of *M* vs QIP, *q*, is obtained for the standard. Since the relationship between *q* and *M* is assumed to be independent of the radionuclide, the same equation can be used to obtain *M* for each of the other nuclides based on the measured *q* for that nuclide as long as the measurements are carried out using a set of standards with cocktail compositions identical to those of the radionuclide being analyzed. By performing this fit of *M* vs *q*, it is possible to make the necessary (small) adjustments to the figure of merit between cocktails which are slightly mismatched in composition, volume, or any other condition that leads to slightly different quenching. Based on the *M* calculated from this equation, the efficiency for that particular nuclide can be obtained for those quenching conditions using the tables generated by EFFY4.

EFFY4 calculations were carried out on a PC-clone equipped with a 90 MHz Pentium processor and 16 MB of RAM. The code was modified to permit very large arrays to be carried through the calculation and the output was modified to print only a two-column file of efficiency vs figure of merit. This modified code was recompiled using a 32 bit compiler and run to generate tables for ^3^H and ^63^Ni with figure of merit increments of 0.0001 through the entire range of observed ^3^H efficiencies. The *β*-spectral distribution for each radionuclide was calculated over intervals of 0.01 keV in each case using the latest available data from the ENSDF database [2, 15] (*E_β_*_max,Ni_ = 66.945 keV + 0.004 keV, *E_β_*_max,H_ = 18.594 keV + 0.008 keV). Typical calculation times of 10 min to 15 min were required to generate each of the tables.

### 3.2 Activity Measurement Results

The general data analysis scheme for any given experiment was as follows:

1. The count rates of all background blanks were averaged.
2. The count rate for each cocktail was corrected for background by subtracting the average integral background rate from the observed gross integral count rate of the cocktail.
3. The counting rates were then decay corrected to a common reference time of 1200 EST August 15, 1995.
4. The corrected count rate for each cocktail was divided by the mass of the added radioactive solution to obtain a massic rate *R_x_* with unit s^−1^ · g^−1^.
5. An average massic count rate, ${\bar{R}}_{x}$, and an average QIP, $\bar{q}$ were obtained for each cocktail.
6. An average efficiency for each ^3^H cocktail was calculated from the known massic activity of the ^3^H standard and the experimental massic count rate for each ^3^H cocktail.
7. Based on each average efficiency, an average figure of merit, ${\bar{M}}_{3H}$, was obtained for that cocktail by looking up the corresponding *M*-value for that average efficiency in the tables generated by EFFY4 for ^3^H.
8. A third-degree polynomial fit of ${\bar{M}}_{3H}$ vs $\bar{q}$ was obtained.
9. From this fit, a figure of merit, ${\bar{M}}_{63_{\text{Ni}}}$, for each ^63^Ni cocktail was calculated from its $\bar{q}$.
10. Using the tables generated by EFFY4, an average efficiency for each ^63^Ni cocktail was obtained. Dividing this efficiency into the average massic count rate for each ^63^Ni cocktail, the massic activity, *C*_A_ could be calculated.

All calculations were performed using a commercially-available spreadsheet program and most operations outlined above were performed automatically with programs using the spreadsheet’s programming language.

As each experiment was performed, the data were analyzed and manipulated to search for a large number of experimental effects. The results of these searches are outlined in the subsections below, as are the uncertainty components associated with those effects. In summary, the experiments performed for this calibration:

- were performed with two LS spectrometers with different operating characteristics (e.g., linear vs logarithmic pulse amplification, different coincident pulse resolving times, detection thresholds, and instrument-specific quench-indicating methods—see Table 2.
- were made with three commercial scintillation fluids having different scintillation fluors and solvent compositions;
- employed matched ^3^H and ^63^Ni cocktails at two different LS cocktail total volumes, nominally 10 mL to 11 mL and 15 mL;
- were made with cocktails having total aqueous content (on a mass fraction basis) that ranged from about *f* = 0.0001 to *f* = 0.125 water (*f* = 0.30 to *f* = 0.38 water in gel phase);
- used cocktails with an acidic content in the aqueous portion that ranged from 1 mol·L^−1^ HCl to less than 0.004 mol·L^−1^ HCl;
- were made with cocktails having variable Ni^+2^ carrier ion concentrations in the aqueous portion that ranged from about 0.4 mg to 100 mg Ni^+2^ per gram of solution;
- used varying quantities of both nitromethane (nitromethane, or 10 % (by volume) solutions of nitromethane in ethanol) and water (or slightly acidic NiCl_2_ carrier solutions) as imposed quench agents to develop the efficiency tracing curves;
- involved a range of LS cocktail counting rates (for both ^3^H and ^63^Ni) from 400 s^−1^ to over 2200 s^−1^;
- incorporated measurements on cocktails ranging in “age” from < 1 d to nearly 90 d (where the age is the time difference between cocktail preparation and measurement time);
- covered efficiency-tracing quenching ranges that corresponded to ^63^Ni detection efficiencies from 0.60 to 0.81, and ^3^H efficiencies of 0.17 to 0.56;
- involved 34 separate efficiency tracing curves each of which consisted of 4 to 9 cocktails that had been independently measured 3 to 9 times; and
- included data on a total of 976 sets of measurements on 55 matched ^63^Ni and ^3^H LS cocktails.

**Fig. 2:**
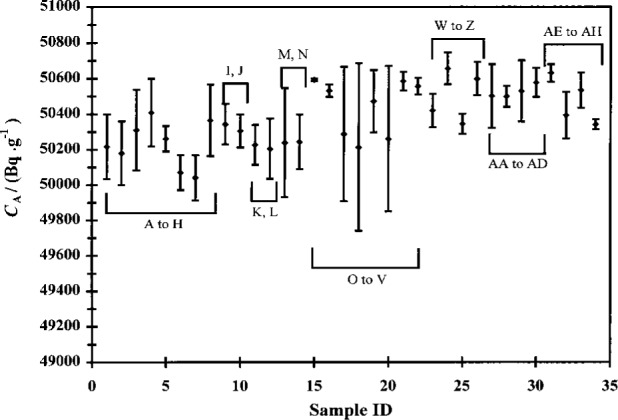
Average massic activities *C*_A_ (in Bq ·g^−1^) of the ^63^Ni cocktails from all 34 experiments performed in this investigation. The uncertainty bars indicate the standard deviation *s* due to reproducibility between the cocktails in the series.

The experimentally-determined massic activities *C*_A_ for each cocktail series are presented graphically in Fig. 2.

### 3.3 Calculation of Certified Massic Activity and Estimation of Uncertainty Components

Based upon our newly acquired knowledge pertaining to sample loading in LS cocktails and examination of the individual efficiency-traced massic activities from each of the cocktails in each series, decisions were made as to which data were to be included in the average for the final massic activity value. Because of the low aqueous fractions of the cocktails and the fact that no attempt was made to match the ^3^H and ^63^Ni cocktails closely in terms of composition, data from experiments “A” to “N” were excluded from the average. Two cocktails each from experiment series “O,” “P,” “U,” and “V” were excluded due to inadvertent errors in mass determinations in the ^63^Ni added to the cocktails. The first cocktail in each of the cocktail series was excluded on the basis of having extremely low water fractions, which resulted in apparently outlying *C*_A_ values. For the same reason, the first two cocktails from series “Q,” “R,” “S,” and “T” were excluded. Finally, none of the gel cocktails were included in the average (see section 3.3.6) and the first cocktail in the two “normal” phase Insta-Gel cocktail series were excluded on the basis of being considered outliers due to their low water fractions. In all, 72 cocktails from 18 efficiency tracing curves were used to arrive at a *C*_A_ value for ^63^Ni.

The mean ^63^Ni massic activity for those 72 cocktails was found to be 50.534 kBq ·g^−1^ ± 0.113 kBq ·g^−1^ at the stated reference time, where the uncertainty is the standard deviation for measurement reproducibility between the 72 cocktails. Likewise, the mean ^63^Ni massic activity found by averaging the means determined in each cocktail series was found to be 50.530 kBq ·g^−1^ ± 0.089 kBq ·g^−1^, where the uncertainty is the standard deviation for activities determined amongst the 18 efficiency curves. Therefore, the certified massic activity *C*_A_ value for this SRM is 50.53 kBq ·g^−1^ ± 0.46 kBq ·g^−1^ as of the reference time, where the stated uncertainty is the standard uncertainty.

**Fig. 3:**
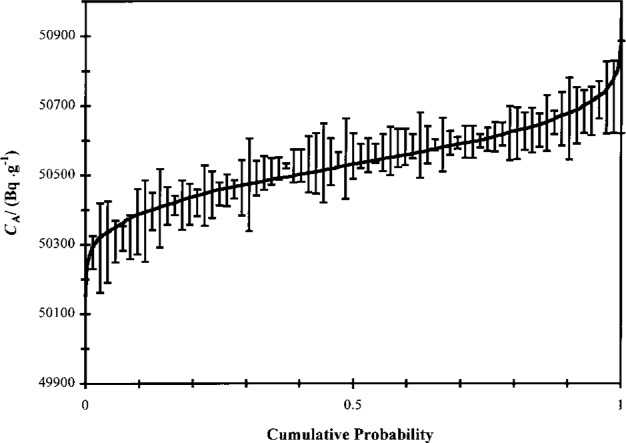
Cumulative probability plot for the data obtained in the present calibration experiments. The uncertainty bars indicate the standard deviation of measurement repeatability for three to seven measurements on a single LS cocktail. The solid line represents random values drawn from a normal distribution with mean *μ* = 50.534 kBq ·g^−1^ and standard deviation *σ* = 0.113 kBq · g^−1^.

**Fig. 4:**
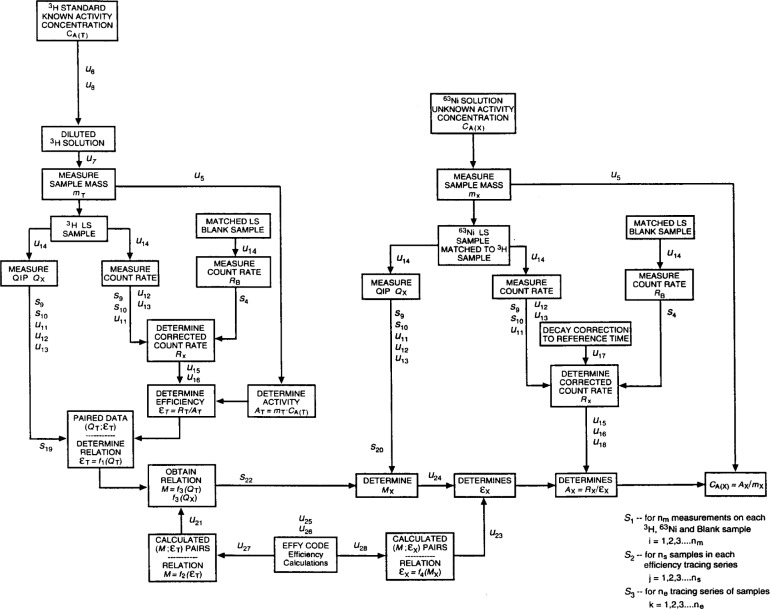
Assumed measurement model for 4*πβ* LS spectrometry of ^63^Ni using ^3^H-standard efficiency tracing as given by the CIEMAT/NIST method. The component uncertainties are identified by *u_i_* and *s_i_* and correspond to the values reported in Table 3.

A cumulative probability plot of the data from all of the calibration experiments is shown in Fig. 3. Although the data appear to be normally distributed, they do not constitute a homogeneous population. In particular, there are three separate and quantifiable contributions of measurement variability to the uncertainty. These are LS measurement *repeatabilit*y for several measurements on a single LS cocktail within a series, *reproducibility* among the different cocktails comprising a series, and *reproducibility* among measurements between series (which comprise a single efficiency curve). Analysis of these data revealed a total of 28 components of uncertainty in applying the CIEMAT/NIST method in this calibration. These are presented in Table 3. The measurement model assumed in the elucidation of the ^63^Ni massic activity and its associated uncertainty components is shown schematically in Fig. 4. Each of the components will now be separately addressed and are referred to by their identifier given in Table 3.

#### 3.3.1 Uncertainty Contributions From Measurement Variability

As stated above, there are three distinct measurement variability components that contribute to the overall uncertainty. The first, *s*_1_, represents the LS measurement *repeatability* on any single cocktail. This was calculated by averaging five (effective degrees of freedom *ν*_eff_ = 4) standard deviations of the mean *s*_m_ calculated from randomly-chosen cocktails from among the 72 cocktails used in the calculation of the central *C*_A_ value and has a magnitude of 0.055 %. Each *s*_m_ had degrees of freedom *ν* = 2 to 5. This uncertainty estimator has an uncertainty itself, equal to about 50 %, which is the relative standard deviation of the five *s*_m_ values used to calculate *s*_1_.

The second measurement variability contribution results from reproducibility among the different LS cocktails within a cocktail series. This was calculated as the standard deviation of the massic activity *C*_A_ for all 18 cocktail series, each with *ν* = 2 to *ν* = 4, giving a *ν*_eff_ = 3 for this uncertainty estimator. This component, *s*_2_ = 0.15 %, is one of the larger uncertainty components. The uncertainty of this uncertainty component, found from the relative standard deviation of the 18 *s*_2_ values, was about 60 %. Since the only difference between each of the cocktails is the amount of quench, this uncertainty component indicates the quenching dependence on the determination of *C*_A_.

The third component, *s*_3_, is the variability between massic activities among the 18 experiments used to calculate the central *C*_A_ value. It is given as the standard deviation of *C*_A_ values obtained from the 18 cocktail series used in the *C*_A_ value calculation and has a magnitude of 0.18 %. This component can be thought of as an indication of the variability due to cocktail composition effects.

Combining these three components in quadrature (*s* = (*Σ s_i_*^2^)^1/2^), it is discovered that the overall contribution of measurement variability to the determination of *C*_A_ is 0.24 %, or roughly only one-fourth of the overall uncertainty.

#### 3.3.2 Background Measurement Variability

The background measurement variability, *s*_4_, was taken as the Poisson “counting error” in 180 determinations of the matched LS background blanks performed during the calibration experiments. This average uncertainty is equal to 0.22 % and leads to an uncertainty in the massic activity *C*_A_ of *s*_4_ = 0.0004 %.

It should be pointed out that although this component can be separated and quantified, it is partially embodied in the measurement variability components. This is because the same variation in the background count rates is also somewhat contained in the variation of gross counting rates of the LS cocktails.

#### 3.3.3 Gravimetric (Mass) Determinations for LS Cocktail

The value taken for this component, *u*_5_, is a canonical value used by this laboratory and is largely due to the inherent uncertainties in the internal balance weights and in the reproducibility in the optical scale of the single-pan microbalance. The magnitude of the uncertainty is *u*_5_ = 0.05 %.

#### 3.3.4 Uncertainty Components Associated With ^3^H Standard

Perhaps the largest contributor to the overall uncertainty in this calibration is due to the uncertainties associated with the ^3^H standard used in the efficiency tracing. There are three components of the uncertainty associated with the ^3^H standard. The first is the certified standard uncertainty for the SRM 4927E ^3^H standard (0.34 %) which must be unfolded from an applied decay correction (0.29 %), leaving 0.18 %, keeping in mind that the components are combined in quadrature. When this is propagated through the calculation of the massic activity, it is found that this leads to an estimate of 0.11 % for *u*_6_.

The second component, *u*_7_, takes into account the gravimetric dilution that was performed on the ^3^H standard. A similar 0.05 % standard uncertainty due to gravimetric determinations applies to this gravimetric dilution and must be considered separately from *u*_5_. This is because this uncertainty is propagated through the ^3^H-standard efficiency tracing by affecting the value of the ^3^H activity used in calculations. When this uncertainty in the dilution is propagated, it leads to a value for *u*_7_ of 0.03 %.

The component, *u*_8_, takes into account the uncertainty in the ^3^H activity that arises from the uncertainty in the ^3^H half-life as it is decayed over 16.95 a. The standard uncertainty of the ^3^H half-life as compiled by ENSDF [15] is 0.46 %, which, when propagated, leads to a value 0.27 % for u_8_. In evaluating this component, it is assumed that timing uncertainties are negligible.

#### 3.3.5 Spectrometer Dependence (*s*_9_)

**Fig. 5:**
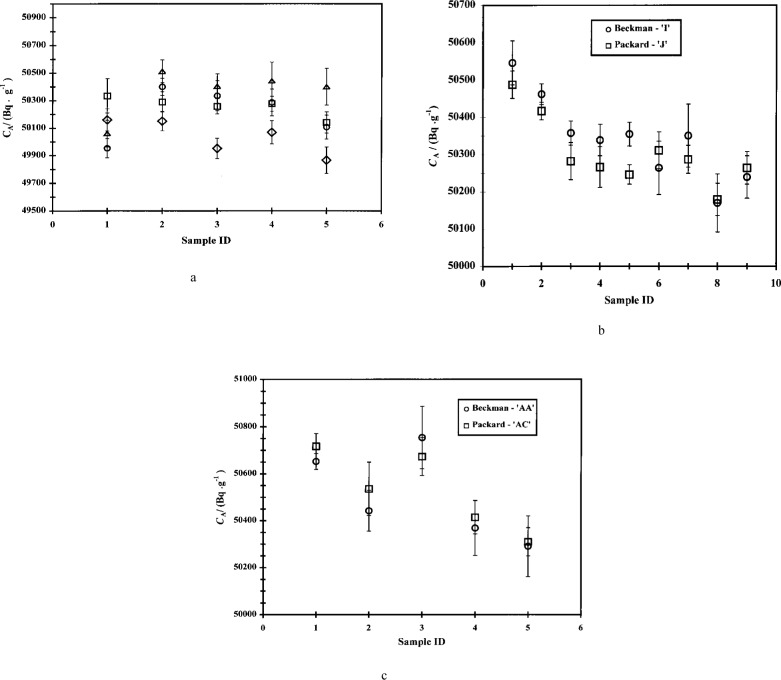
Plots of efficiency-traced massic activity *C*_A_ (in Bq · g^−1^) for ^63^Ni experiments using the Beckman and Packard spectrometers with two different commercially-available scintillation fluids, Ready Safe and Ultima Gold. Referring to Table 1, Fig. 5a contains data from experiments “A” (circles), “E” (squares), “G” (diamonds), and “H” (triangles), all with aqueous fraction of cocktail *f* = 0.001; Fig. 5b contains data from experiments “I” and “J”; and Fig. 5c contains data from experiments “AA” and “AC.” Water fractions in Figs. 5a and 5c were nominally 3 % and 10 %, respectively. The uncertainty bars represent measurement repeatability for the same LS cocktail with typical degrees of freedom *ν* = 5.

As discussed above, two commercial spectrometers, both with different operating characteristics, were used in the calibration experiments. A comparison of the characteristics of both spectrometers is given Table 2. Plots of three sets of data in which the aqueous fraction of the total LS cocktail *f* was similar for all cocktails in the series but in which the two spectrometers were used are shown in Fig. 5. The data in Fig. 5a appear to indicate a slight dependence on the spectrometer used, especially for the cocktails which used Ultima Gold. Based on these observations, a series of *t*-tests was performed on these data, which indicated that while the differences in means between the Ready Safe cocktails were not statistically significant, those between the Ultima Gold cocktails were. One possible explanation for these observations lies in the compositions of the various cocktails. For the cocktails in Fig. 5a, the aqueous fraction *f* is at a minimum, nominally 0.1 %. As will be discussed below, this water fraction has been shown to be too low to give reliable results. The reliability of measurements made using cocktails with an aqueous fraction of less than *f* = 0.05 is questionable at best. Further, it is shown below that Ultima Gold appears to be more sensitive to water fraction effects than Ready Safe.

Two more experiments involving Ultima Gold were performed and those data are plotted in Figs. 5b and Fig. 5c. The data from these experiments exhibit much better agreement between the two spectrometers, with the *t*-tests indicating no statistical significance between the means of the data from each spectrometer at the 95 % confidence level. The water fractions of the cocktails shown in Figs. 5b and Fig. 5c are nominally 3 % and 10 %, respectively, and could be considered to be sufficiently high as to provide reliable counting measurements. Because of the extremely low water fractions of the LS cocktails of the data shown in Fig. 5a, that set of data can be considered anomalous and it can be concluded that there is little or no variability in the activity measurements due to the spectrometer used.

It may be desirable, however, to attempt to quantify any possible effect due to spectrometer differences. One way to do this is to take the ratio of the mean massic activity of the 13 cocktail series which used one spectrometer and the mean massic activity of the 17 cocktail series using the other spectrometer. When this is done, it is found that this ratio is equal to 0.9990 ± 0.0047, where the given uncertainty is the propagated standard uncertainty on the ratio.

Because of the way in which the experiments were performed and the large amount of data, any effect due to differences in spectrometer would be completely embodied in the measurement variabilities outlined in Sec. 3.3.1.

#### 3.3.6 Scintillant Dependence (*s*_10_)

**Fig. 6:**
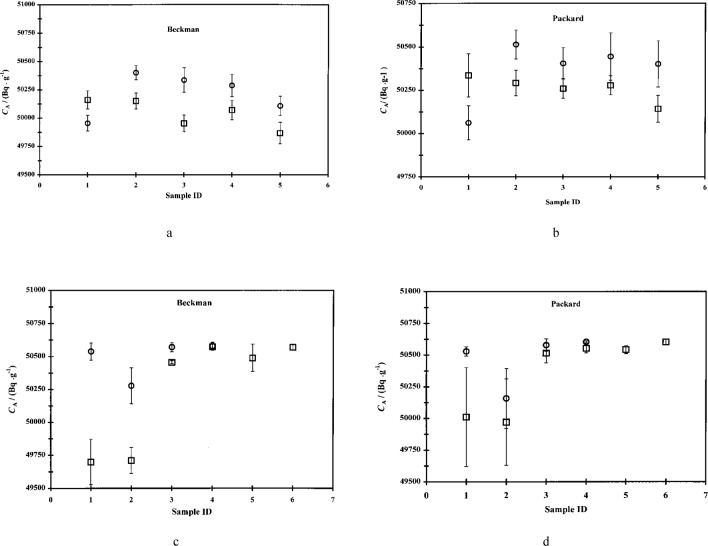
Plots of efficiency-traced massic activity *C*_A_ (Bq ·g^−1^) for ^63^Ni experiments involving two different commercially-available scintillation fluids, Ready Safe (circles) and Ultima Gold (squares), in the Beckman and Packard spectrometers. Fig. 6a contains data from experiments “A” and “G,” Fig. 6b from experiments “E” and “H,” Fig. 6c from “O” + ”P” and “Q” + “R,” and Fig. 6d from “S”+”T” and “U” + ”V.” Water mass fractions Figs. 6a and 6b were 0.1 %, while those for Figs. 6c and 6d varied from 0.3 % to 11 %. Uncertainty bars represent measurement repeatability for a single cocktail with typical degrees of freedom *ν* = 9.

Although three commercially-available scintillation fluids were used in the course of these experiments, there are only sufficient data to make a comparison between Ready Safe and Ultima Gold. Even those data are further complicated by cocktail composition effects. Plots comparing Ready Safe and Ultima Gold with different cocktails series using both spectrometers are shown in Fig. 6. While an initial examination of these plots may show large dependencies on the scintillator fluid used, consideration of the cocktail composition casts doubt on this interpretation. In the case of Figs. 6a and 6b, the aqueous fraction of the cocktail is only 0.001. As has been alluded to previously, and will be discussed at length below, this amount of water is insufficient to provide reliable measurements. One particularly interesting aspect of these plots is the consistently low activity value obtained for the first cocktail, which corresponds to minimum quenching. This effect is apparent in the data obtained from both spectrometers, although the absolute magnitudes of the efficiency-traced activities is somewhat lower in the Beckman relative to the Packard.

Inasmuch as many routine LS measurements are made with cocktails with such low water fractions, an evaluation of the uncertainty of the measured activity is appropriate. Taking the means of the activity values with both the Ready Safe and Ultima Gold cocktails in the Beckman, we obtain an average massic activity of 50.13 kBq · g^−1^. The median of these two sets of values is also 50.13 kBq · g^−1^. Using this value, we then calculate a relative standard deviation s of 0.12 % for those 10 cocktails. Similarly, the Packard data have a mean activity of 50.31 kBq ·g^−1^ and from this we calculate a relative standard deviation *s* of 0.17 %. It cannot be overemphasized, however, that cocktails with such low water content should be avoided whenever possible. This point will be further discussed below.

Turning to the data in Figs. 6c and Fig. 6d, we find a significant difference between the efficiency-traced massic activities of the first two cocktails relative to the others in both spectrometers. Again, this is most likely due to cocktail composition effects. The first two cocktails only have nominally *f* = 0.003 and *f* = 0.03, respectively. Although there are only two Ready Safe data values with water fractions greater than 3 % for each spectrometer, the data that do exist appear to suggest that there is no uncertainty introduced by the interchange of Ready Safe and Ultima Gold. Even if there were an effect due to differences in the scintillants, the effect would again be embodied in the measurement variability.

In order to estimate an overall uncertainty in the efficiency-traced *C*_A_ due to use of these two scintillants without regard to cocktail composition, ratios of the mean massic activities of the cocktails obtained with one scintillant to that using the other (similar to what was done in estimating the spectrometer dependence in Sec. 3.3.5) can be evaluated. When this is done, it found that this ratio is 1.0008 ± 0.0048 where the uncertainty is the standard uncertainty on the ratio. Again, because of the experimental design, any effect of employed scintillant is completely embodied in the measurement variability.

The final scintillant effect studied was the difference between phases (“normal” and “gel”) in scintillants that form viscous, opaque gels when the water fraction is high (> 25 % or so). At low water fractions, the scintillator is less viscous and is transparent, although there is generally an intermediate water fraction region in which the two phases (water and scintillant) are immiscible and as such the cocktail cannot give reliable results. The final sets of cocktails (“AE” to “AH”) were prepared to determine if any difference could be observed in the efficiency-traced massic activities of the ^63^Ni between the two phases.

**Fig. 7:**
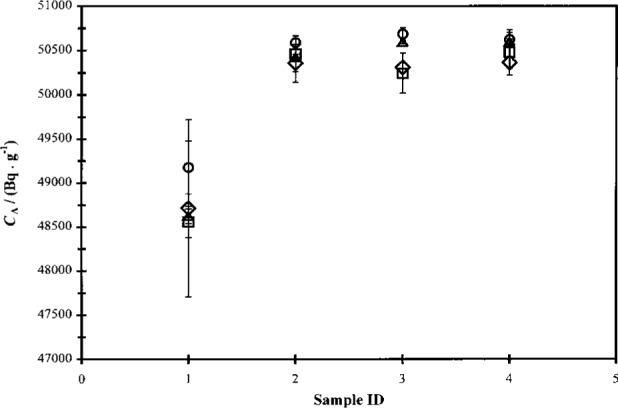
Plot of efficiency-traced massic activity *C*_A_ (in Bq ·g^−1^) for ^63^Ni experiments using Insta-Gel XF in either “normal” (cocktails “AE” and “AG”) or “gel” (cocktails “AF” and “AH”) states in both the Beckman (circles and squares, respectively) and Packard (triangles and diamonds, respectively) spectrometers. The uncertainty bars represent the measurement repeatability for activity determinations for the same LS cocktail over 6 independent measurements of the massic activity in the same spectrometer.

The data are plotted in Fig. 7, and as can be seen, there is again an anomaly in the least-quenched cocktails. The surprising result is that this effect is the same in both the “normal” and “gel” phases; that is, that even though the “gel” cocktails have a large (about 25 %) amount of added water, the least-quenched cocktail in that phase still gives a low activity result. One possible key to understanding what is happening may lie in the uncertainties associated with these cocktails. There is a systematic trend in all four of the measurements in that the decay-corrected count rates *in all cases* are dropping with each successive count cycle, suggesting some sort of cocktail instability. This is plausible when one considers that in the “normal” cocktails, the water fraction is about 1.5 %—well below the critical value of 5 %, which we found to be the lower limit for obtaining consistent results. However, this still does not fully explain the behavior of the “gel” series. The lowest water fraction in the “gel” series was about 30 %, which is well within the limits quoted by the manufacturer [16] as the water fraction range that gives reliable results in the “gel” phase. Nontheless, the present results suggest that the cocktail is not stable. The reason for this is unknown and may remain a mystery due in part to the proprietary nature of the chemistry of these commercial scintillants.

A statistical *t*-test on the data from the last three cocktails in both series suggest that there is indeed a statistical difference in the means of the efficiency-traced massic activities between the “normal” or “gel” phases. Neglecting the first cocktail in both the “normal” and “gel” and combining the remaining 12 values of the massic activity (6 LS cocktails times two spectrometers), a mean value of 50.48 is obtained. The mean of the 3 “normal” cocktails (in 2 spectrometers) is 50.58 kBq · g^−1^, while the mean of the 3 “gels” is 50.37 kBq · g^−1^. The uncertainty of activity measurements made with the scintillant in the two phases can be estimated by considering the difference between the two means for the “gel” and “normal” samples compared to the mean obtained using the extant set of 12 activity determinations. This yields a difference of 0.20 %, which is multiplied by a coefficient of 0.89 (for an unbiased estimate of the standard deviation from the range of two measurements) to give an estimate of the standard uncertainty of 0.18 % on the two phases.

#### 3.3.7 Effects of LS Cocktail Age and Solution Stability (*u*_11_)

**Fig. 8:**
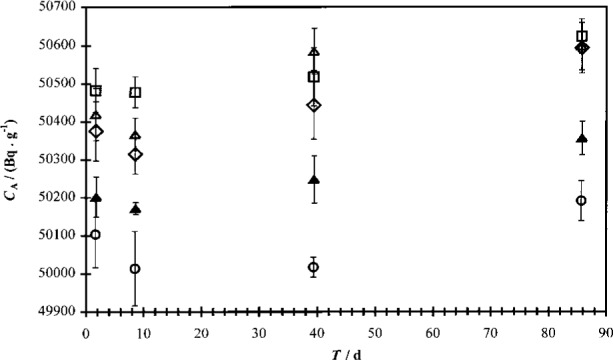
Plot of efficiency-traced massic activity *C*_A_ (in Bq · g^−1^) for ^63^Ni (ampoule 30, Experiments “A” to “D”) as a function of the time *T* (in d) between LS cocktail preparation and median of count interval of five 20 min cycles. The uncertainty bars denote the one standard uncertainty interval for measurement repeatability across five cycles. The aqueous fraction *f* of the total cocktail mass was 0.001 in Ready Safe. Data were acquired on the Beckman spectrometer. The data series are denoted “R1” to “R5” (circles, squares, triangles, diamonds, and filled triangles, respectively), where “R1” is the least-quenched cocktail.

The effect of cocktail stability was studied by counting the first set of prepared cocktails over a period of 86 days (cocktails “A” to “D”). These data are plotted in Fig. 8. For each cocktail series, a linear fit was performed in order to estimate the effect of the time interval between source preparation and measurement on the traced massic activity. Taking an average of 4 days between preparation and completion of counting (typical for the combination of two spectrometers) for all but the least quenched cocktail (“R1”), an average increase in the massic activity of 0.032 % is observed for those cocktails over this time interval. It should be pointed out that this study was performed on cocktails with very low aqueous fractions (*f* = 1 × 10^−5^) and as such, the magnitude of this effect may or may not be the same in cocktails with higher water fractions. In fact, they would probably be expected to be lower because of the noticeable increase in cocktail stability at higher water fractions. This effect is partially embodied in the measurement variability.

#### 3.3.8 LS Cocktail Composition Dependence (*u*_12_)

As pointed out in the Introduction, one of the properties of ^63^Ni that makes it so important in the field of radionuclide metrology is its low *β*-endpoint energy, which makes it somewhat more sensitive to subtle effects in the measurement process that would not be apparent in higher-energy *β*-emitters. One of these effects is the influence of the mass fraction *f* of water in the LS cocktail. One early indication that *f* was important was the magnitude of the measurement variabilities of the cocktails. An examination of Fig. 2 shows that the measurement uncertainties between cocktails is in general larger in experiments “A” to “V” (where the water fraction was nominally *f* = 0.001 to *f* = 0.03) then in Experiments “W” to “AH” (in which *f* was maintained above 0.05). The value of *f* = 0.05 is also the minimum recommended by at least one of the scintillant manufacturers [17].

**Fig. 9:**
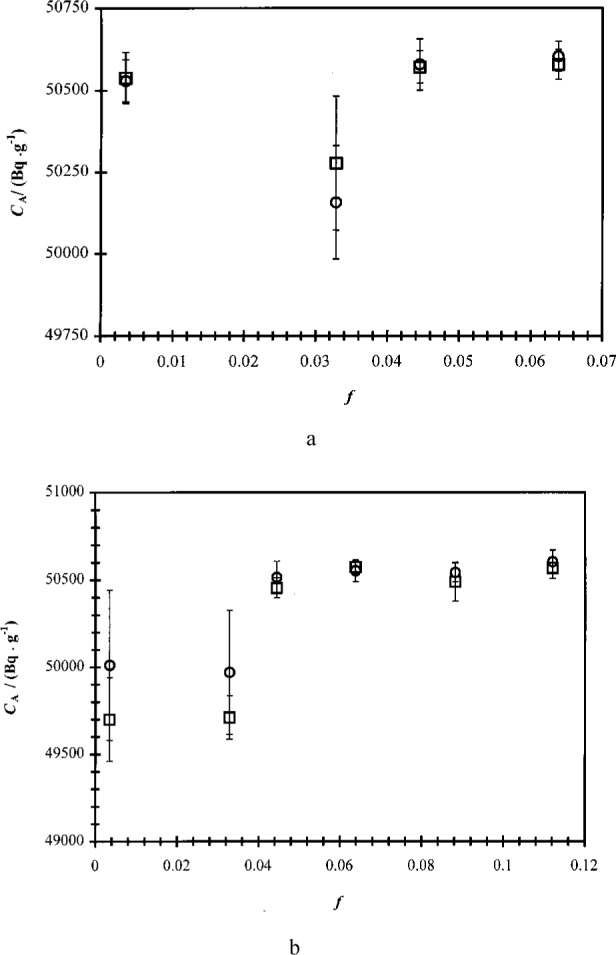
Efficiency-traced massic activity *C*_A_ (in Bq ·g^−1^) of ^63^Ni standard solution as a function of water mass fraction *f* of the LS cocktail. Data are presented for both the Packard (circles) and Beckman (squares) spectrometers using Ready Safe [Fig. 9a] and Ultima Gold [Fig. 9b] as the scintillants. A constant cocktail volume of nominally 10 g was maintained for all cocktails (cocktails series “O” to “V”). The uncertainty bars denote the measurement repeatability of a single cocktail over three to five independent measurements of the massic activity.

Cocktail stability (i.e., the measurement variability or replication) is not the only manifestation of these cocktail composition effects, however. Experiment series “O” to “V” were specifically designed to investigate the effect of water fraction not only on the measurement variability, but also on the magnitude of the efficiency-traced massic activity itself. The results of this investigation are presented in Fig. 9. It is clear from these data that the two scintillation fluids employed exhibit vastly different behavior from each other. With the exception of the cocktail with *f* ≈ 0.00035, all of the *C*_A_ values from cocktails prepared using Ready Safe are identical within their respective measurement uncertainties. At this point it is unknown why that single value is an apparent outlier.

In cocktails prepared with Ultima Gold, the behavior is a bit more erratic. Those cocktails with *f* lower than 0.05 exhibit much larger variability between measurements on the same cocktail. Furthermore, this variability suddenly and drastically decreases when *f* is increased over 0.05. This lower variability appears to be maintained throughout the region between 0.05 and about 0.12, although it should be expected to once again increase as the water fraction approaches the cocktail loading limits of the particular cocktail (*f* = 0.15 to *f* = 0.20, depending upon the scintillator and the temperature).

The other striking effect observed for this scintillant is the apparent difference in the massic activity between cocktails with water mass fraction *f* < 0.05 and those with mass fractions *f* < 0.05. The mean *C*_A_ for the low *f*(< 0.05) cocktails is 49.85 kBq · g^−1^ ± 0.26 kBq · g^−1^, while that of the higher-*f* cocktails is 50.55 kBq · g^−1^ ± 0.06 kBq · g^−1^—a difference of 1.4 %. Ultima Gold was allegedly formulated to be capable of relatively high cocktail loading (up to 20 % water), but there is no mention in any manufacturer’s literature of a *lower* limit on the amount of water that needs to be present in a cocktail in order to obtain reliable results. The danger with this effect is that had the same aqueous fractions been used in all of the experiments as in the first three experiments, the reported value would have been incorrect by about 1.4 % and there would have been no way to know otherwise.

A full analysis of the uncertainties introduced due to total aqueous fractions, as well as mismatches in water compositions between the ^3^H and ^63^Ni cocktails, is given by Collé [8, 9]. In the present calibration study, however, any uncertainty due to cocktail mismatches are completely embodied in the measurement variability. Uncertainty due to using the “wrong” water content was eliminated by using only *C*_A_ values obtained from cocktails having at least 4 % water fraction in the final average.

The associated uncertainty component and its effect on the efficiency-traced massic activity can be estimated by a fit of the massic activity vs. the aqueous fraction *f* of the cocktail. This fit revealed an uncertainty of 0.06 % for the composition range used in the calibration experiments and is partially embodied in the LS measurement variabilities.

#### 3.3.9 LS Cocktail Mass (or Volume) Dependence (*u*_13_)

The topic of total cocktail volume dependence of the efficiency-traced massic activity is extensively treated in Ref. [7]. A linear fit of *C*_A_ vs total cocktail volume (with constant cocktail composition) can be obtained, indicating a 0.04 % uncertainty across the volume range of the cocktails used in computing the central value. This uncertainty is also partially embodied in the uncertainties due to measurement variability.

#### 3.3.10 Effect of LS Cocktail Mismatch Between ^3^H and ^63^Ni Cocktails (*u*_14_)

This topic has been dealt with extensively by Collé [8, 9]. For the purposes of estimating the uncertainty due to mismatches between the ^3^H and ^63^Ni cocktails, it is necessary to compare the QIP *q* for the “matched” cocktails and calculate how this difference in *q* affects the final calculated *C*_A_. Examination of the extant data set indicates that for allegedly “matched” ^3^H and ^63^Ni cocktails, *q* can differ by as much as 3 % to 4 %. Fortunately, this effect is entirely embodied in the LS measurement variabilities.

#### 3.3.11 Spectrometer Timing Effects

Two uncertainty components can be associated with timing effects involving the spectrometers themselves. The first component, *u*_15_, is the standard uncertainty of the livetime counting intervals of 0.07 %, which was obtained from an estimate of 0.1 % for each instrument and is reduced by a factor of 2 for the two spectrometers.

The other component, *u*_16_, accounts for the uncertainty in the deadtime that is not compensated for by the spectrometers themselves. This is evaluated [18] by assuming that the deadtime correction is 10 % and that the spectrometer is capable of correcting for the deadtime to within 10 ms to 15 ms for an uncertainty of 0.1 % for each spectrometer. This is reduced by a factor of $\sqrt{2}$ (for two spectrometers), giving a value of 0.07 %, which leads to a standard uncertainty contribution of 0.04 % to *C*_A_ due to *u*_16_.

#### 3.3.12 Decay Corrections for ^63^Ni from Measurement Time to Reference Time (*u*_17_)

In performing decay corrections from the midpoint of an LS counting interval to the reference time, the uncertainty in *C*_A_ can arise from two sources. The first, the uncertainty in determining the time difference Δ*T* over which the decay correction is made, is due exclusively to the uncertainty in the start times of the counting intervals as reported by the respective spectrometers. This is estimated to be ± 1 min over 50 d (1.4 × 10^−3^ %), which was the longest Δ*T* over which any decay correction was made. Propagating this through the calculation of *C*_A_ indicates that the contribution from this uncertainty to *u*_17_ is very small, on the order of 10^−8^.

The dominant contribution, however, is the standard uncertainty in the half-life of ^63^Ni, which is equal to 1.4 × 10^−2^ [15]. When this uncertainty in the half-life is propagated through the calculation of *C*_A_, an estimate of *u*_17_ = 1 × 10^−3^ % is obtained.

#### 3.3.13 Determination of Quench-Indicating Parameters (QIP)

Measurement of *q* for each quenched cocktail of both the ^3^H and ^63^Ni is crucial for the determination of the ^63^Ni counting efficiencies. The *q* values determined for ^3^H are used to develop the equations that relate the figure of merit *M* to *q*, which is in turned used to calculate a new *M* for each of the ^63^Ni cocktails based upon the measured ^63^Ni *q* values. The relative standard uncertainties for *s*_19_ and *s*_20_ are 0.34 % for both the ^3^H and ^63^Ni and are based upon repeatability with four cocktails chosen at random from among the extant data set, each with between three and seven determinations of *q*. These uncertainty components each have an uncertainty of 60 % associated with them. When propagated through the *C*_A_ calculations, the magnitudes of *s*_19_ and *s*_20_ are found to be 0.09 %. These two uncertainties are also partially embodied within the measurement variabilities discussed above.

#### 3.3.14 Uncertainties Associated With Calculations Performed in the CIEMAT/NIST Method

In this particular application of the CIEMAT/NIST method, there are eight identifiable uncertainty components which affect the uncertainty of *C*_A_. Two of them, identified as *u*_21_ and *u*_23_ in Table 3 are due to the step sizes used in the EFFY4 calculations. The input to EFFY4 allows the user to define the increment in *M* to be used in the calculations. Because of the different *E_β_*_max_ of ^3^H and ^63^Ni, the change in efficiency *ε* for a given change in figure of merit *M*, d*ε*/d*M*, is different in each case. Increments of 0.0001 in *M* were used to generate the two lookup tables used for this calibration. For the quenching range used in these experiments, the average ratio of d*ε*/d*M* for ^63^Ni compared to ^3^H is about 0.59, which can be termed a relative efficiency factor *S*. For ^3^H, this step size uncertainty is 0.008 % which results in a value of *u*_23_ ≈ 0.002 % on application of the factor *S*.

Another uncertainty introduced by the use of EFFY4 is that due to the uncertainties in the *E_β_*_max_ of the two radionuclides. In the case of ^3^H, the uncertainty of *E_β_*_max_ is 0.04 %. The effect of *E_β_*_max_ for ^3^H on the *C*_A_ for ^63^Ni can be estimated from a re-calculation of the table of *ε* vs *M* when the *E_β_*_max_ for ^3^H is increased by 0.04 %. From this, one finds that the mean value of *C*_A_ changes by 0.09 %, which is taken as the value of *u*_27_. Similarly, the uncertainty in *E_β_*_max_ for ^63^Ni is 0.006 % and the value of *C*_A_ changes by 0.0024 %, leading to the value of *u*_28_.

Two more uncertainty components, for *s*_22_ and *s*_24_, are introduced by the fits of the measured *q* for ^3^H vs the ^3^H figure of merit *M*_3H_ obtained from the EFFY4 calculations or the fit of the ^63^Ni figure of merit *M*_63Ni_ vs the calculated ^63^Ni efficiency *ε*_63Ni_. In the case of *s*_22_, the standard deviations of the fitting parameters for third-order fits from four independent curves of *M* vs *q* were typically 0.12 % and resulted in a relative standard uncertainty contribution to *C*_A_ of 0.02 %.

Evaluation of *s*_24_ was a bit more subtle because it enters the uncertainty assessment only in an indirect manner. A fit of ^63^Ni efficiency vs *M* is actually never performed in this application of the CIEMAT/NIST method. Rather, this fit enters into *s*_23_ through the variability in the *q* determinations and the ∂*M*/∂ (*q*) sensitivity. That is, a change in the ^63^Ni activity Δ*ε*_63Ni_ can be expressed as

$$\Delta \varepsilon_{63\text{Ni}}=\Delta \left(q\right)\cdot \frac{\partial \varepsilon_{\text{Ni}}}{\partial M}\cdot \frac{\partial M}{\partial \left(q\right)},$$

which results from a corresponding change Δ(*q*) in the QIP determination, and where $\frac{\partial \varepsilon_{\text{Ni}}}{\partial M}\;\text{and}\;\frac{\partial M}{\partial \left(q\right)}$ and can be estimated from the slopes of the fits of *ε* vs *M* for ^63^Ni and *M* vs *q* for ^3^H. From this, one can estimate

$$s_{24}=s_{22}\cdot \frac{\partial \varepsilon_{\text{Ni}}}{\partial M}\cdot \frac{\partial M}{\partial \left(q\right)},$$

which is the value of component *s*_24_ using the value of component *s*_22_.

#### 3.3.15 Other Uncertainties Affecting Efficiency Calculation

Two components are identified which rely solely on evaluations by other investigators. The first, the effect of ionization quenching assumptions on the efficiency calculations, is an evaluation of the uncertainty introduced by the assumption of the particular model used by EFFY4 to account for ionization quenching effects. This is essentially an evaluation of the uncertainty of the function *Q*(*E*) and the effect of that uncertainty on the calculated *C*_A_ value. The magnitude of this component, *u*_25_, is based on the mean of two values cited in Refs. [19, 20] and has a magnitude of 0.1 %.

The second component, the effect of phototube asymmetry on the efficiency calculations is likewise evaluated from the mean of the values cited in Refs. [19, 20] and leads to a value of *u*_26_ of 0.14 %. This component accounts for efficiency losses that could be present if the responses of the PMTs are assumed to be different by 10 %.

#### 3.3.16 Impurity Analysis

The ^63^Ni solutions were radionuclidically very pure. The solutions were independently examined for possible photon-emitting impurities using high-purity intrinsic germanium detectors [21]. None were detected. Lower limits *L*, in terms of the ratio of the number of photons emitted per second to the ^63^Ni activity in units of Bq, at various energy regions, were:

$$\begin{array}{l} \begin{array}{l} \text{in}\;\text{the}\;\text{region}\;12\;\text{keV}-88\;\text{keV},\\ L<5\times 10^{-6}s^{-1}\cdot {\text{Bq}}^{-1}; \end{array}\\ \begin{array}{l} \text{in}\;\text{the}\;\text{region}\;96\;\text{keV}-507\;\text{keV},\\ L<2\times 10^{-6}s^{-1}\cdot {\text{Bq}}^{-1}; \end{array}\\ \begin{array}{l} \text{in}\;\text{the}\;\text{region}\;515\;\text{keV}-1456\;\text{keV},\\ L<8\times 10^{-7}s^{-1}\cdot {\text{Bq}}^{-1};\;\text{and} \end{array}\\ \begin{array}{l} \text{in}\;\text{the}\;\text{region}\;1456\;\text{keV}-1900\;\text{keV},\\ L<3\times 10^{-7}s^{-1}\cdot {\text{Bq}}^{-1}. \end{array} \end{array}$$

The relative standard uncertainty in the efficiency-traced massic activity due to photon-emitting impurities was estimated to be equal to the estimated limit of detection for the impurities; that is, that the relative standard uncertainty is 100 %. Propagating these uncertainties gives for the uncertainty *u*_18_ a value of 4 × 10^−4^ %.

### 3.4 Other Effects Evaluated but not Included in the Uncertainty Budget

#### 3.4.1 Comparison of Solutions M4 and M5: Results Over 27 Years

Because all three of the ^63^Ni SRMs issued by NBS/NIST over the past 27 years are gravimetrically related to a common solution source, it was possible to check the accuracy of the dilution factors, or alternatively, to check the stability of the solution over this time interval. The relationship amongst the various SRMs provided the information necessary to experimentally determine the half-life of ^63^Ni from radioactive decay for the first time [1]. Solutions originating from M1 and M2 (see Fig. 1) were gravimetrically diluted to give two new solutions (denoted M4 and M5 respectively) and were counted in the LS spectrometers as outlined above. The results were normalized by their respective dilution factors to solution M3, from which the ampoules of the present SRM were prepared. The results are tabulated in Table 4.

A rather complete analysis of the previous NBS/NIST calibrations, along with a comparison of the present calibration, is given by Collé and Zimmerman [22]. By adjusting the former results to reflect the latest available nuclear decay data, as well as reassessing the uncertainty estimation of the former results, it was found that the massic activities *C*_A_ of all three calibrations agree to within 0.3 % when the appropriate decay corrections are made. Considering that the standard uncertainties of each of the calibrations are of the order of 1 %, this shows remarkable consistency in the measurement capabilities of NBS/NIST.

#### 3.4.2 Differences Between SRM Ampoules

**Fig. 10:**
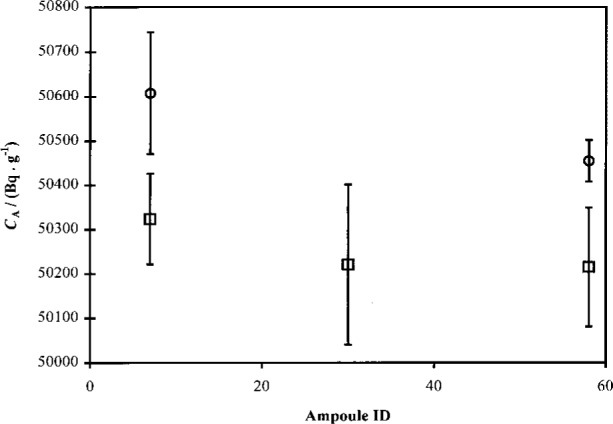
Comparison of massic activities *C*_A_ (in Bq · g^−1^) of ^63^Ni obtained from experiments with three different ampoules, 7, 58, and 30, chosen at random from the 60 ampoules of SRM 4226C prepared. The squares indicate measurements performed using relatively low (< 3 %) aqueous fraction in the LS cocktail, while the circles indicate higher (≈ 10 %) aqueous fractions. The uncertainty bars signify the one standard uncertainty interval for measurement reproducibility amongst all cocktails with similar LS cocktail composition prepared with ^63^Ni from the same ampoule.

Because the experiments performed for this calibration utilized ^63^Ni solution from 4 randomly-chosen ampoules from among the 60 that were prepared, it should be possible to look at variations in the efficiency-traced massic activity of the ^63^Ni solutions in those ampoules. No two investigations, however, used the same LS cocktail composition (identical scintillator, identical aqueous fraction, carrier composition, quenching agent, etc.). Thus direct comparisons between all four ampoules are not possible. The best comparison that can be made is shown Fig. 10. In that plot, solutions from ampoules 7, 30, and 58 are compared for both high (≈10 %) and low (≤ 3 %) LS water fractions. Only low water fraction data are available for ampoule 30. The low aqueous fraction data are complicated by much lower (≈ 0.1 %) fractions used in ampoules 58 and 30 relative to ampoule 7 (3 %). It is clear from Fig. 10 that there is good agreement between ampoules when the LS water fractions are nearly equal. Moreover, with only two exceptions, all of the data agree with each other within the measurement uncertainties. Based on these data, however sparse, it can be concluded that there is no additional uncertainty component due to the choice of solution ampoule. Furthermore, any small effect that might be present is completely embodied in the measurement variabilities.

## 4. Conclusion

A new set of ^63^Ni solution standards has been prepared by the Radioactivity Group of NIST and was issued as NIST SRM4226C. The certified massic activity for these standards is 50.53 kBq ·g^−1^ ± 0.92 kBq · g^−1^ at a reference time of 1200 EST August 15, 1995, where the uncertainty is the expanded uncertainty (coverage factor *k* = 2) based on the evaluation of 28 different uncertainty components which were evaluated on the basis of an exhaustive number (976) of LS counting measurements investigating over 15 variables. By far the largest uncertainty in the determination of *C*_A_ arises from the uncertainty of the decay correction of the ^3^H standard. It alone comprises roughly one-fourth of the overall uncertainty. Measurement variability (comprised of repeatability on a single LS cocktail, reproducibility between cocktails in a given cocktail series, and reproducibility between different cocktail series with different quench curves) accounts for another quarter of the uncertainty. Much of the remaining half of the uncertainty is due to various steps involved in the application of the CIEMAT/NIST method.

A large number of LS cocktail measurement effects were investigated, including that of mass fraction of water in the cocktails. It was found that reproducibility between measurements on the same cocktail was poor for cocktails when the mass fraction of the water was less than 5 %. Furthermore, these studies gave the first indication that the carrier ion concentration in that water fraction may also play an important role.

As low-energy *β*-emitters become increasingly used as radionuclidic standards, an understanding of cocktail effects such as those associated with total cocktail mass or volume, total water mass fraction, and ion concentrations are crucial. The experiments performed in this calibration are serving as a starting point for larger, more systematic studies of how these variables interact and influence the final results. We intend to continue searching for and evaluating these effects and encourage other laboratories, especially other standards laboratories, to do likewise.

## Figures and Tables

**Table 1:** Summary of LS cocktail compositions used in the preliminary and calibration experiments. In the table, each experiment trial is identified with an alphabetic identifier (ID). The remaining categories are: the originating ampoule (Amp.) used to prepare the ^63^Ni cocktails; the commercial scintillant (Scin.) employed in the ^63^Ni and ^3^H cocktails, where RS = Ready Safe, UG = Ultima Gold, and GEL = Insta-Gel XF; the spectrometer used (Spec.) for the given trial, where B = Beckman and P = Packard; the number of cocktails *n*_c_ prepared for each experiment; the range of cocktail ages (Age) in days between the time of sample preparation and midpoint of the LS counting interval; LS cocktail water mass fraction (*f*); and the masses of employed scintillant (*m*_s_), imposed quenching agent (*m*_q_), added water (*m*_w_), added Ni^+2^ carrier solution (*m*_c_), and either the ^63^Ni or ^3^H solution (*m*_x_)

ID	Amp.	Scin.	Spec.	*n*_c_	Age (d)	*f*	*m*_s_ (g)	*m*_q_ (g)	*m*_w_ (g)	*m*_c_ (g)	*m*_x_ (g)
A	30	RS	B	5	1–2	0.001	15	0–0.4	0	0	0.01–0.02
B	30	RS	B	5	8–9	0.001	15	0–0.4	0	0	0.01–0.02
C	30	RS	B	5	39–40	0.001	15	0–0.4	0	0	0.01–0.02
D	30	RS	B	5	85–86	0.001	15	0–0.4	0	0	0.01–0.02
E	30	UG	P	5	1–2	0.001	15	0–0.4	0	0	0.01–0.02
F	30	UG	P	5	8–9	0.001	15	0–0.4	0	0	0.01–0.02
G	30	UG	B	5	4–5	0.001	15	0–0.4	0	0	0.01–0.02
H	30	RS	P	5	4–5	0.001	15	0–0.4	0	0	0.01–0.02
I	7	UG	B	9	0.2–2	0.03	14.5	0–0.4	0.5	0	0.02–0.03
J	7	UG	P	9	2.2–4	0.03	14.5	0–0.4	0.5	0	0.00–0.03
K	58	RS	B	6	0.1–1.5	0.003–0.014	10	0–0.06	0–0.08	0–0.08	0.02–0.06
L	58	RS	P	6	3.3–4.5	0.003–0.014	10	0–0.06	0–0.08	0–0.08	0.02–0.06
M	M5	RS	B	5	0.1–1.7	0.003–0.014	10	0–0.06	0–0.08	0–0.08	0.01–0.07
N	M4	RS	B	5	0.1–1.7	0.003–0.014	10	0–0.06	0–0.08	0–0.08	0.01–0.05
O1	30	RS	B	6	0.2–1.6	0.003–0.11	8.8–10	0	0.02–1.2	0.02–1.2	0.01–0.02
P1	30	RS	B	6	6.1–7.1	0.003–0.11	8.8–10	0	0.02–1.2	0.02–1.2	0.01–0.02
Q	30	UG	B	6	3.8–5.4	0.003–0.11	8.8–10	0	0.02–1.2	0.02–1.2	0.01–0.02
R	30	UG	B	6	7.2–8.1	0.003–0.11	8.8–10	0	0.02–1.2	0.02–1.2	0.01–0.02
S	30	UG	P	6	0.2–1.6	0.003–0.11	8.8–10	0	0.02–1.2	0.02–1.2	0.01–0.02
T	30	UG	P	6	6.1–7.1	0.003–0.11	8.8–10	0	0.02–1.2	0.02–1.2	0.01–0.02
U^a^	30	RS	P	6	3.8–5.4	0.003–0.11	8.8–10	0	0.02–1.2	0.02–1.2	0.01–0.02
V^a^	30	RS	P	6	7.2–8.1	0.003–0.11	8.8–10	0	0.02–1.2	0.02–1.2	0.01–0.02
W	58	RS	B	5	0.1–2.4	0.09–0.2	10.08–11.04	0	0.9–1.9	0.9–1.9	0.02
X	58	UG	B	5	0.1–2.4	0.08–0.20	10.08–11.04	0	0.9–1.9	0.9–1.9	0.02
Y	58	RS	P	5	4.2–7.0	0.09–0.2	10.08–11.04	0	0.9–1.9	0.9–1.9	0.02
Z	58	UG	P	5	4.2–7.0	0.08–0.20	10.08–11.04	0	0.9–1.9	0.9–1.9	0.02
AA	48	UG	B	5	2.8–4.0	0.1	10.5	0–0.75	0–1.2	0–1.2	0.02
AB^b^	48	UG	B	4	2.8–4.0	0.1	10.5	0–0.75	0–1.2	0–1.2	0.02
AC	48	UG	P	5	0.1–1.5	0.1	10.5	0–0.75	0–1.2	0–1.2	0.02
AD^b^	48	UG	P	4	0.1–1.5	0.1	10.5	0–0.75	0–1.2	0–1.2	0.02
AE^c^	7	GEL	B	4	0.1–1.7	0.015–0.11	6.5–11.5	0	0–5.5	0.02	0.02
AF^d^	7	GEL	B	4	0.1–1.7	0.30–0.38	6.5–11.5	0	0–5.5	0.02	0.02
AG^c^	7	GEL	P	4	1.9–3.5	0.015–0.11	6.5–11.5	0	0–5.5	0.02	0.02
AH^d^	7	GEL	P	4	1.9–3.5	0.30–0.38	6.5–11.5	0	0–5.5	0.02	0.02

a Problems in determining the masses of cocktails 27, 30, and 31 caused these particular cocktails to be excluded from the overall average.

b The fraction of added Ni^+2^ carrier solution that comprised the aqueous fraction of the cocktail was varied, but the overall aqueous fraction of the cocktail remained constant.

c “Normal” Cocktails—the first cocktail gave spurious results and was not included in the average.

d “Gel” Cocktails—the first cocktail gave spurious results and was not included in the average.

**Table 2:** Characteristics of the NIST LS spectrometers employed in the calibration of ^63^Ni SRM 4226C

Characteristic	System B	System P
LS spectrometer model	Beckman LS7800	Packard Tri-carb A2500TR
Operating mode	Sum-coincidence	Sum-coincidence
Photomultiplier tubes	Hamamatsu R331-05	Hamamatsu R331-08
Operating temperature	Ambient	Ambient
Coincidence resolving time	22 ns	18 ns
Sum-coincident pulse amplification	Logarithmic	Linear
Pulse resolving time	5 μs to 33 μs (variable with pulse height)	12μs (fixed)
Spectral analog-to-digital converter (ADC) capacity	1000 channels	2048 channels
Nominal conversion gain (energy per channel)	Variable (with logarithmic energy)	≈1 keV
Detection threshold (nominal)	≤ 1 keV	≤ 1 keV
Live-time determination method (and uncertainty)	Gated oscillator (scaled) (± 0.1 %)	Gated oscillator (scaled) (± 0.1 %)
Quench indicating parameter (QIP)	Horrocks number (*H*)	Transformed Spectral Index of the External Standard (*tSIE*) (proprietary)
External (-ray source for QIP determination	^137^Cs	^133^Ba
(and location)	(side)	(bottom)

**Table 3:** Standard uncertainty components for the ^63^Ni massic activity *C*_A_ of SRM 4226C, calibrated by 4*πβ* LS spectrometry with ^3^H-standard efficiency tracing

Uncertainty component and descriptor, followed by propagated uncertainties^a^	Uncertainty type (A or B)^a^ and comments	Relative uncertainty (%)
*s*_1_, LS measurement variability	A; standard deviation of the mean *s*_m_ in *C*_A_ for repeatability of LS measurements on any one LS cocktail (*ν*_eff_ = 4 effective degrees of freedom); typical value obtained from 72 independent determinations of *s*_m_ (each with *ν* = 2 to 5 degrees of freedom); the “relative uncertainty (Δ) of the uncertainty estimator” (relative standard deviation of *s*_m_ values) is 50 %	0.055
*s*_2_, LS cocktail (quench dependence) variability	A; standard deviation *s*_2_ in *C*_A_ for reproducibility among cocktails (differently quenched) within a single efficiency tracing curve (*ν*_eff_ = 3); obtained from 18 independent determinations of *s*_2_ (each with *ν* = 2 to 4); Δ (relative standard deviation of *s*_2_ values) is 60 %	0.15
*s*_3_, LS efficiency tracing (cocktail composition variability	A; standard deviation *s*_3_ in *C*_A_ for reproducibility between 18 efficiency tracing curves for cocktails of differing composition (*ν* = 17)	0.18
*s*_4_, background measurement variability	A; standard deviation *s*_4_ (Poisson “counting error,” with magnitude 0.22 %) in background from 180 determinations on matched LS blanks.	0.004 (and PE)^b^
*u*_5_, gravimetric (mass) determinations for LS cocktails	B; estimated standard uncertainty of mass for any one LS cocktail	0.05 (and PE)^b^
*u*_6_, ^3^H standard primary calibration	B; for a standard uncertainty of 0.18 % from NIST calibration	0.11
*u*_7_, ^3^H standard gravimetric dilution	B; estimated standard uncertainty of gravimetrically-determined dilution factor	0.03
*u*_8_, ^3^H standard decay correction	B; for a standard uncertainty in half-life of 0.46 % for decay over 16.95 a; uncertainty in timing is negligible	0.27
*s*_9_, LS spectrometer dependence	A; ratio of mean *C*_A_ between 13 efficiency tracing curves obtained with one spectrometer and 17 curves with the other was 0.9990 ± 0.0047	WE^b^
*s*_s10_, scintillator dependence	A; ratio of mean *C*_A_ between 15 curves obtained with one scintillator and 11 curves with another was 1.0008 ± 0.0048	WE^b^
*u*_11_, LS cocktail stability age dependence	B; estimated standard uncertainty of fit of relation between *C*_A_ and cocktail age (time between cocktail preparation and measurement)	0.03 % (and PE)^b^
*u*_12_, LS cocktail composition dependence	B; estimated standard uncertainty of systematic relation between *C*_A_ and aqueous mass fraction in LS cocktail	0.06 % (and PE)^b^
*u*_13_, LS cocktail mass (or volume) dependence	B; estimated standard uncertainty of systematic relation between *C*_A_ and cocktail volume	0.04 % (and PE)^b^
*u*_14_, mismatch of LS cocktail composition in ^3^H, ^63^Ni and blank cocktails	B; estimated standard uncertainty of about 3 % to 4 % based on comparison of quench indicating parameters for matched cocktails	WE^a^
*u*_15_, livetime determinations for LS counting time intervals	B; estimated standard uncertainty of 0.1 % for each of 2 spectrometers	0.07
*u*_16_, uncorrected deadtime counting effects	B; estimated standard uncertainty corresponding to 1 % of correction (for each of two spectrometers) divided by $\sqrt{2}$	0.04
*u*_17_, for ^63^Ni from measurement time to common reference time	B; for estimated standard uncertainties in timing (0.001 %) and ^63^Ni half-life (1.4 %) for decay over intervals < 0.2 a	0.001 (and PE)^b^
*u*_18_, radionuclidic impurities	B; none detected; estimated standard uncertainty corresponding to the detection limit for the photonic-emission rate	0.0004
*s*_19_, determination of quench indicating parameter (QIP) *q*, for ^3^H	A; for a relative standard deviation *s_q_* of 0.34 % (based on repeatability with 4 cocktails each with *n* = 2 to 6); Δ (relative standard deviation of the four *s*_18_ values) is 60 %	0.09 (and PE)^b^
*s*_20_, determination of *q* for ^63^Ni	A; for a relative standard deviation *s_q_* of 0.34 % (based on repeatability with four cocktails each with *n* = 2 to 6); Δ (relative standard deviation of the four *s*_19_ values) is 60 %	0.09 (and PE)^b^
*u*_21_, precision of ^3^H efficiency versus figure of merit (*M*) calculations	B; calculational step sizes	0.008
*s*_22_, fit of relation between ^3^H QIP and calculated *M*	A; for a standard deviation of 0.12 % on the fit for 4 independent *M* vs *q* curves	0.02
*u*_23_, precision of ^63^Ni efficiency vs *M* calculations	B; calculational step sizes	0.002
*s*_24_, fit of relation between calculated *M* and ^63^Ni efficiency	A; for a standard deviation of 0.006 % from the fit of the relation between the calculated efficiency and *M*	0.002
*u*_25_, effect of ionization quenching assumptions on efficiency calculations	B; estimated standard uncertainty	0.1
*u*_26_, effect of asymmetry in phototube responses on efficiency calculations	B; estimated standard uncertainty of 0.2 % for this effect divided by $\sqrt{2}$ for two spectrometers	0.14
*u*_27_, effect of ^3^H *E_β_*_max_ on efficiency calculations	B; for an estimated standard uncertainty of 0.04 % in *E_β_*_max_	0.09
*u*_28_, effect of ^63^Ni *E_β_*_max_ efficiency calculations	B; for an estimated standard uncertainty of 0.006 % in *E_β_*_max_	0.0024
*u*_c_, combined standard uncertainty^a^	quadratic combination of all components of uncertainty; *u*_c_ = (Σ*_i_u_i_*)^1/2^ for *i* = 1 to 28 with *u_i_* = *s_i_* for type A components	0.46
*U* = *k* × *u*_c_, expanded uncertainty^a^	for *k* = 2, which is assumed to correspond to a confidence level of about 90 to 95 %	0.92

a Refer to accompanying text for definition of terms.

b The relative uncertainty for this component is wholly (WE), or in part (PE), embodied in the relative standard uncertainties of components *s*_1_, *s*_2_, *s*_3_.

**Table 4:** Comparison of efficiency-traced massic activities $C_{{A,}^{63}\text{Ni}}$ of solutions M3, M4, and M5 (see Fig. 1)

Solution Identifier (cocktail series)	$C_{{A,}^{63}\text{Ni}}/\text{kBq}\cdot g^{-1}$
M3 (K,L)	50.23 ± 0.11
M4 (M)	50.24 ± 0.15
M5 (N)	50.24 ± 0.31

a All massic activities are normalized by the appropriate gravimetric dilution factors to solution M3, which represents the present calibration. See Table 1 for compositions of LS cocktails of the appropriate cocktail series for each solution. The uncertainties are the standard uncertainties corresponding to the variability in the massic activity due to reproducibility between LS cocktails in a given cocktail series.

## Footnotes

1 The term “massic activity” is that preferred by the International Organization for Standardization (ISO) for the quantity obtained by dividing the total amount of radioactivity in a sample divided by the sample mass. The quantity is expressed in the International System of Units (SI) as Bq · g^−1^.

2 Certain commercial equipment, instruments, or materials are identified in this paper to foster understanding. Such identification does not imply recommendation by the National Institute of Standards and Technology, nor does it imply that the materials or equipment identified are necessarily the best available for the purpose.

3 CIEMAT/NIST is an acronym for Centro de Investigationes Energetica, Medioambientales y Technologicas and the National Institute of Standards and Technology, which are the respective national standards laboratory of Spain and the United States. Researchers from the two institutions collaboratively developed the efficiency tracing method which bears the names of those laboratories.
